# Morphometry and Immunoexpression of Metalloproteinase 2 and Its Inhibitor in the Fibrotic and Non-Fibrotic Grafted Kidney—Digital Analysis

**DOI:** 10.3390/biomedicines14030524

**Published:** 2026-02-26

**Authors:** Dagmara Szypulska-Koziarska, Ewa Kwiatkowska, Martyna Opara-Bajerowicz, Aleksandra Wilk

**Affiliations:** 1 Department of Histology and Embryology, Pomeranian Medical University in Szczecin, Powstańców Wielkopolskich Al. 72, 70-111 Szczecin, Poland; aleksandra.wilk@pum.edu.pl; 2 Department of Nephrology, Transplantology and Internal Medicine, Pomeranian Medical University in Szczecin, Powstańcow Wielkopolskich Al. 72, 70-111 Szczecin, Poland; ewa.kwiatkowska@pum.edu.pl (E.K.); martyna.oparabajerowicz@gmail.com (M.O.-B.)

**Keywords:** kidney, fibrosis, biopsies, metalloproteinase, TIMP

## Abstract

**Background**: Metalloproteinases (MMPs), together with their tissue inhibitors (TIMPs), regulate the extracellular matrix (ECM) in various tissues. MMP-2 and TIMP-2 maintain this process in renal tissue. An imbalance in the MMP-2/TIMP-2 ratio alters the abundance and proportions of specific extracellular matrix components, leading to kidney fibrosis. We aimed to assess differences in the morphometric parameters of the kidney and the immunohistochemical (IHC) expression of MMP-2 and TIMP-2 in kidney biopsies according to their fibrotic state. **Methods**: The histological slides were scanned using the 3DHISTECH Pannoramic MIDI II Scanner, and the resulting digital images of the sections were analyzed using Pattern Quant software; the morphometric analyses were performed with the Slide Viewer application. **Results**: In the current manuscript, we have investigated the significant enlargement of the diameter of the urinary space and renal corpuscle, as well as the reduced height of the epithelial lining of the proximal and distal convoluted tubules, of grafted kidneys with fibrosis when compared to the non-fibrotic kidneys. Moreover, we have noticed a rising MMP-2/TIMP-2 ratio in the immunohistochemical reaction in the renal tissue of fibrotic grafted kidneys in comparison to healthy kidneys. **Conclusions**: These results suggest that the MMP-2/TIMP-2 ratio, together with the lower inhibition of MMP-2, may promote an increased extracellular matrix remodeling, which accompanies the development of fibrosis.

## 1. Introduction

Numerous clinical conditions lead to chronic kidney disease (CKD), and each case is associated with fibrosis [1]. Moreover, the deterioration of kidney graft function results from graft fibrosis, followed by secondary tubular atrophy. It is known that renal fibrosis is primarily caused by the accumulation and activation of myofibroblasts and fibroblasts within the renal interstitium, where these cells are surrounded by an extracellular matrix (ECM). Sun et al. indicate that renal myofibroblasts emerge de novo during kidney fibrosis, share a characteristic phenotype with fibroblasts, and may be induced by cellular stress [2].

Homeostasis is one of the crucial factors that regulate the proper function of organs, including the kidneys. Metalloproteinases (MMPs) and their tissue inhibitors (TIMPs) are enzymes that regulate the turnover of extracellular matrix components. This large group of zinc-dependent enzymes shares similar activities and structure [3,4,5,6]. The functions of MMPs include the modulation of cell–ECM adhesion, cell–cell interactions, cell survival, proliferation, and motility. The MMPs are initially synthesized in an inactive form, and the activation of these proenzymes can be induced intra- or extracellularly [7,8]. They are crucial for tissue remodeling and play an important role in many renal diseases, both acute and chronic [3,7]. Matrix MMP-2 is abundantly expressed in the kidney, cleaving components of the glomerular and tubular basement membrane and degrading other ECM structures of the renal tissue.

Enzymes that can inhibit the active form of MMP-2 are TIMPs. The increased expression of TIMPs has been associated with glomerulosclerosis. TIMP-2 is involved in the activation of pro-MMP-2 [7]. Although TIMP-2 is a major inhibitor of MMP-2, it also facilitates the activation of pro-MMP-2 via membrane type 1 metalloproteinase (MT1-MMP), which is anchored to the cell membrane [9]. TIMP-2 induces G1 cell cycle arrest by binding to human endothelial cells through integrin α3/β1. This early G1 cell cycle arrest during cellular injury may serve as a biomarker for predicting acute kidney injury in vivo [10]. In renal tissue, MMP-2 expression varies depending on its localization along the nephron. For example, MMP-2 and TIMP-2 are highly expressed in epithelial, mesangial, and proximal tubular cells. Their expression is also detected near the tubular basement membrane and within the interstitial tissue [11]. There is limited data regarding the correlation between various kidney diseases and changes in activity and/or the immunoexpression of MMP-2 and/or TIMP-2; however, Rysz et al. have correlated decreased levels of both MMP-2 and TIMP-2 with diabetic nephropathy [12]. Early diabetic nephropathy was proven to be associated with an increase in TIMP-2 immunoexpression, together with a decrease in MMP-2 immunoexpression [13]. Moreover, a deficiency in TIMP-2 activity was related to hypertension and renovascular remodeling [3,9], whereas MMP-2 overexpression and increased activity have been correlated with renal interstitial fibrosis [14], CKD, tubular atrophy or fibrosis [15], acute kidney injury [16], lupus nephritis, and glomerulosclerosis [14]. Some authors have reported that several kidney diseases are associated with an imbalance in the MMP-2/TIMP-2 ratio, including diabetic nephropathy [13], renal cell carcinoma [17,18], and obstructive nephropathy [7,19].

Chronic kidney disease results from persistent renal parenchymal damage or loss. The irreversible decrease in the number of functioning nephrons leads to a gradual inability of the kidneys to maintain homeostasis over weeks to years. The rate of such progression varies according to the underlying pathology. The progression of CKD is driven more by secondary maladaptive hemodynamic and metabolic changes than by the underlying disease itself [20].

In cases of worsening kidney function, a renal biopsy is performed to assess the current state of renal tissue, as many diseases affect glomerular morphology. These include focal segmental glomerulosclerosis, an extensive loss of renal mass, diabetes, oligomeganephronia, reflux nephropathy, obesity, sleep apnea, and unilateral renal agenesis [21].

Our study aimed to assess differences in kidney morphometric parameters, as well as the immunoexpression of MMP-2 and TIMP-2, in kidney biopsies depending on the degree of fibrosis.

## 2. Materials and Methods

### 2.1. Biopsies Protocols

All patients (*n* = 44) enrolled in this research provided written informed consent prior to participation. The study was conducted in accordance with the Declaration of Helsinki and the Declaration of Istanbul, and the protocol was approved by the Ethics Committee of Pomeranian Medical University, Szczecin, Poland, under the study protocol—KB-0012/23/18 (05FEB2018). In the current research, we analyzed 44 biopsies from kidney transplant recipients (*n* = 44), in 22 of which the fibrosis was confirmed (*n* = 22), while the remaining 22 showed no histological evidence of fibrosis. The inclusion criteria were: formal consent, time since kidney transplantation (at least 12 months), and at least a dual immunosuppression consisting of mycophenolate mofetil or mycophenolate sodium combined with a calcineurin inhibitor. The exclusion criteria were: no formal consent, kidney transplantation in time shorter than 12 months, and an immunosuppression regimen based on mTOR inhibitors. The characteristics of the patients are included in Table 1. The biopsies were performed for clinical indications, such as proteinuria or rising serum creatinine. The study group was divided into subgroups, the control and the fibrotic, based on the presence or absence of chronic changes on the kidney biopsy assessed under a microscope by a pathologist.

### 2.2. Histological Analysis

**Figure 1:**
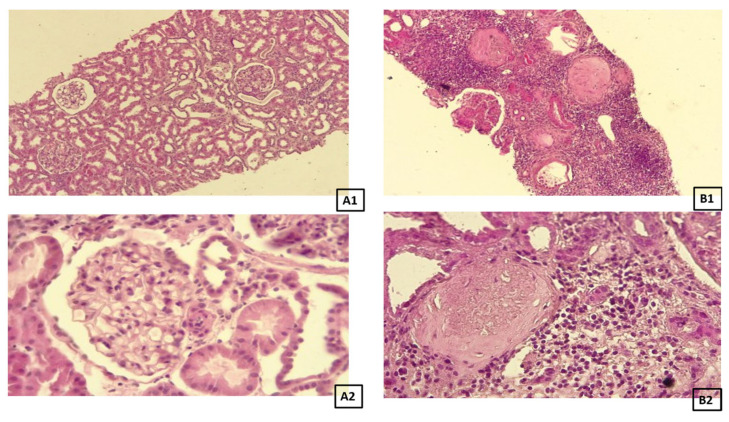
Representative picture of the H&E staining in the control group (**A1**: objective magnification ×20, **A2**: objective magnification ×40) and fibrotic group (**B1**: objective magnification ×10, **B2**: objective magnification ×40) under the light microscope.

**Figure 2:**
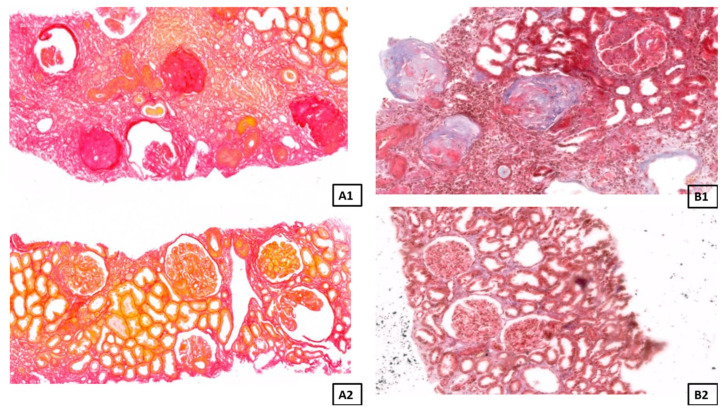
Representative picture of the Picro Sirius Red staining (**A1**,**A2**) in the fibrotic group (**A1**: objective magnification ×20) and in the control group (**A2**: objective magnification ×15.8), and the Masson Trichrome staining (**B1**,**B2**) in the fibrotic group (**B1**: objective magnification ×20), and in the control group (**B2**: objective magnification ×15.8) under a light microscope.

Human kidney biopsies were fixed in 4% buffered formaldehyde, dehydrated in a graded series of ethyl alcohol and xylene, and then embedded in paraffin. Next, paraffin blocks containing renal tissue were cut into 3 µm thick sections. For staining using standard methods, the sections were deparaffinized in xylene, then rehydrated with the use of a graded ethyl alcohol series. Achieved sections were stained with four different staining methods: hematoxylin and eosin (H&E) to visualize the general structure and architecture of the tissue (Figure 1), orcein to observe the elastic fibers, and Masson Trichrome and Sirius Red to visualize collagen fibers (Figure 2). All histochemical reactions were performed according to the manufacturer’s protocols.

The slides were scanned using the 3DHISTECH Pannoramic MIDI II Scanner (Sysmex Polska Sp. z o.o., Warsaw, Poland). The resulting digital images of the sections were analyzed using Pattern Quant software v 2.3 (3DHISTECH Kft., Budapest, Hungary).

### 2.3. Morphometry

**Figure 3:**
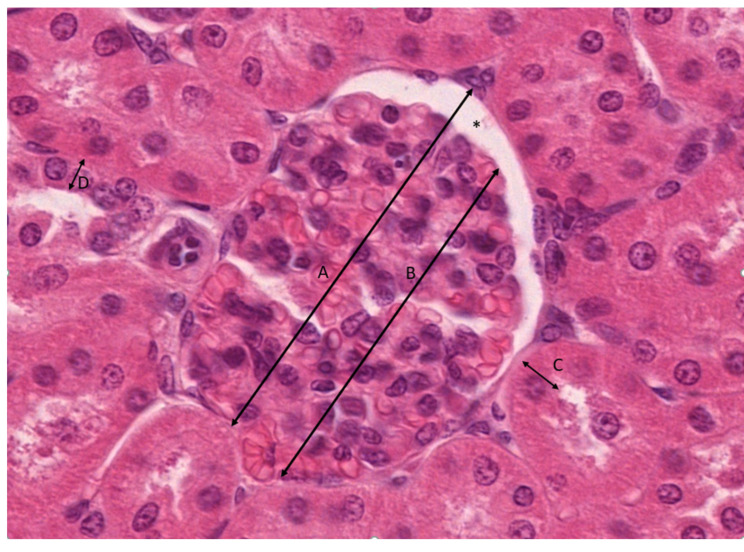
Representative picture of the morphometric method with the use of the Slide Viewer (under 40× objective magnification). The asterisk (*) represents a urinary space width.

The diameter of the renal corpuscle and the glomerulus was measured on the H&E sections using the ruler tool in the Slide Viewer application software v 2.5 (Slide Viewer, 3D Histech, Budapest, Hungary). In each group, more than 150 measurements were taken for each tested variable. The width of the urinary space (*) was calculated as the difference between the renal corpuscle diameter (A) and the glomerular diameter (B). The height of the epithelial lining of proximal (C) and distal (D) convoluted tubules was measured on the H&E sections using the ruler tool. All measurements were recorded in millimeters under 40× objective magnification. Glomerular diameter measurements are illustrated in Figure 3.

### 2.4. Immunohistochemical Analysis

**Figure 4:**
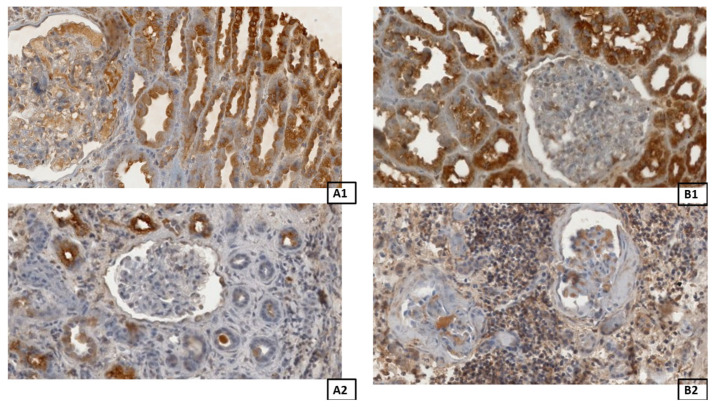
Representative picture of the randomly chosen area of immunoexpression for MMP-2 in the control group (**A1**) and the fibrotic group (**A2**), and the immunoexpression for TIMP-2 in the control group (**B1**) and the fibrotic group (**B2**). Light microscope under 40× objective magnification.

To detect the expression of MMP-2 and TIMP-2, the following antibodies were applied: mouse monoclonal anti-TIMP-2 at 1:250 (sc-53630; Santa Cruz Biotechnology, Inc., Santa Cruz, CA, USA) and mouse anti-TIMP-2 at 1:250 (sc-21735; Santa Cruz Biotechnology, Inc., Santa Cruz, CA, USA). All antibodies were diluted with Diluent (Agilent Dako EnVision, Hovedstaden, Denmark). Slides were deparaffinized in three changes of xylene and rehydrated in a graded series of ethanol and distilled water. For antigen retrieval, the slides were immersed in a 0.01 M citrate buffer at pH 6.0 and subjected to microwave heating for 10 min. Endogenous peroxidases were inhibited through incubation with the use of Dual Endogenous Enzyme Block (Agilent Dako EnVision, Hovedstaden, Denmark) for 10 min. Then, sections were incubated with the above antibodies for 30 min at room temperature. Afterwards, sections underwent a 30-min incubation with labeled polymer (Labeled Polymer HRP; Agilent Dako EnVision, Hovedstaden, Denmark), followed by a 10-min incubation with a substrate–chromogen complex containing 3,3′-diaminobenzidine (Agilent Dako EnVision, Hovedstaden, Denmark), resulting in the formation of a brown precipitate at the antigen site. Negative controls were prepared by excluding the primary antibodies. After counterstaining with hematoxylin (Sigma-Aldrich, Poznań, Poland), the specimens were mounted with a mounting medium. Before each incubation, sections were washed twice in PBS for five minutes, followed by a five-minute TBS rinse. Each incubation was carried out in a humid chamber at room temperature. Staining procedures were conducted according to the manufacturer’s instructions. Representative images of MMP-2 and TIMP-2 in the control and fibrotic groups are shown in Figure 4.

**Figure 5:**
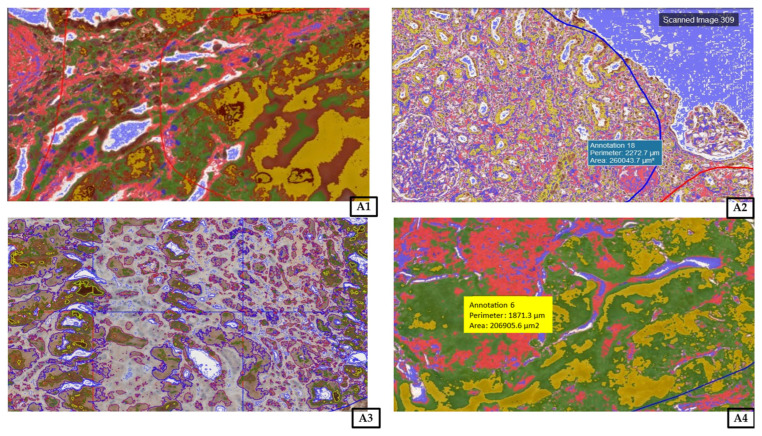
Representative picture of the randomly chosen area of four visible strengths of immunoexpression of the analyzed enzymes. Cell Quant creates a map and marks each observation based on the strength of the immunostaining of the protein: blue indicates negative expression (−); red indicates weak expression (+); green indicates strong expression (++); and yellow indicates very strong expression (+++). Objective magnification as follows: (**A1**): ×43; (**A2**): ×10.7; (**A3**): ×15.7; (**A4**): ×55.

Upon the completion of the reaction, the slides were assessed under a microscope and with LAS software version 4.4 (Leica DM500B, Wetzlar, Germany). To investigate the renal expression of MMP-2 and TIMP-2, all samples were analyzed using Cell Quant software version 2.3 (3DHistech Kft., Budapest, Hungary), which detected four levels of staining intensity and generated area and object intensity measurements of IHC products (Figure 5). The levels of staining were classified based on the intensity of staining. Staining intensity was categorized as follows: blue, negative (−); red, weak (+); green, strong (++); yellow, very strong (+++). A total area of 40,199.66 mm^2^ and 32,279.42 mm^2^ of renal tissue was analyzed for MMP-2 and TIMP-2 immunohistochemical expression, respectively.

### 2.5. Statistical Analysis

All analyses were performed in Stata 18. Continuous data were presented as medians (Me) with interquartile ranges (IQR), since the Shapiro–Wilk test indicated that most results were not normally distributed. Therefore, the Mann–Whitney U test and the Kruskal–Wallis test were applied to assess statistical significance. To evaluate whether there is any correlation between the results, Spearman’s rank correlation test was applied. Statistical significance was set at *p* < 0.05.

## 3. Results

### 3.1. Morphometry

#### Renal Corpuscle, Glomerulus, Urinary Space

A total of 150 measurements for each parameter, which are the renal corpuscle diameter (A), glomerulus diameter (B), and height of the epithelial lining of the proximal (C) and distal (D) convoluted tubules, were taken in both the control and fibrotic groups. Detailed results and correlations between variables are shown in Table 2.

The total positive immunoexpression area for MMP-2 was lower in the fibrotic group than in the control group, and a similar trend was observed for TIMP-2. The MMP-2/TIMP-2 ratio was 0.51 in the control group and 0.78 in the fibrotic group (Table 3).

We observed that the area of strong positive immunoexpression (++) for MMP-2 is significantly higher in the control group when compared with fibrotic grafts (Table 4).

The correlation analysis revealed that, in the control group, the weak positive immunoexpression (+) of MMP-2 was associated with both strong positive (++) and negative (−) immunoexpression. In the fibrotic group, weak positive (+) expression correlated with strong (++), very strong (+++), and negative (−) expression, while strong positive (++) expression was associated with very strong (+++) and negative (−) expression. Furthermore, very strong positive (+++) expression correlated with negative (−) expression (Table 4).

The strong positive immunoexpression (++) for TIMP-2 in the control group was significantly higher when compared with the fibrotic group (Table 5).

As far as the correlations are concerned, in the control group, we observed that weak positive immunoexpression (+) correlates with strong positive immunoexpression (++), and strong positive immunoexpression (++) correlates with very strong positive immunoexpression (+++) (Table 5).

In the fibrotic group, no correlations were observed.

## 4. Discussion

Glomerular hypertrophy, reflected by an increased glomerular diameter, is associated with numerous human diseases. Therefore, our initial analysis focused on morphometric parameters. The results clearly demonstrate that, in grafted kidneys with fibrosis, the diameter of the renal corpuscle was significantly greater compared with grafted kidneys without fibrotic lesions. This enlargement was accompanied by a significant expansion of the urinary (Bowman’s) space, whereas the glomerular diameter itself did not increase proportionally. Specifically, fibrotic biopsies exhibited a significant increase in urinary space compared with the control tissue.

Spearman’s correlation analysis in control kidneys revealed a very strong positive correlation between the diameters of the renal corpuscle and the glomerulus, but only a very weak correlation between the renal corpuscle and the urinary space. A similar pattern was observed in fibrotic biopsies; however, the strength of the correlation was moderate rather than strong. Interestingly, a weak negative correlation between the glomerular diameter and urinary space was identified exclusively in the fibrotic group.

The association between enlarged Bowman’s space and glomerular hyperfiltration suggests that the dilation of the Bowman’s space may result from an increased hydrostatic pressure gradient across the glomerular capillaries. According to the literature, such dilation may reduce the urinary space pressure, thereby permitting the maintenance of a high transcapillary hydrostatic pressure gradient [22]. We have met some difficulties with the available literature on our current topic. There is very limited data describing the morphometrical parameters in grafted human kidneys with and without fibrosis. For example, Tobar et al. [22] have noticed a significant enlargement of the glomerulus area and glomerulus volume, as well as a significant increase in the Bowman’s space and volume in obese patients, in contrast to the control group. Nevertheless, similar results were presented by Kotyk et al.; however, this was in rats only, not humans [21].

Further current analysis revealed that the height of the epithelial lining of both the proximal convoluted tubule and the distal convoluted tubule was significantly lowered in fibrotic kidneys when compared with the control biopsies. Similarly, Tabor et al. [22] observed this in obese patients. In this case, the results would be consistent with our findings in this area. Taking into account the enlargement of the renal corpuscle and the urinary space, the fact that the proximal tubular lumen volume is also increased suggests that the Bowman’s space dilation was insufficient to normalize the Bowman’s space pressure and that the increased pressure was transmitted distally [22]. In addition to the damage induced by this growth-related mechanical stress, the increase in the Bowman’s space pressure may directly affect glomerular epithelial cells, and this is probably the explanation for the results that we have achieved.

Despite increasing interest in extracellular matrix remodeling, the immunoexpression of MMP-2 and TIMP-2 in human kidney biopsies remains insufficiently characterized. Most available studies focus on urinary or serological markers, or on mRNA expression levels, without accounting for potential posttranslational modifications, including gene silencing or downregulation, which may substantially influence protein activity.

In contrast, detailed immunohistochemical analysis allows for the direct assessment of protein localization and distribution within renal tissue compartments, providing more precise insight into tissue conditions and pathological remodeling. Our current approach offers a more specific evaluation by incorporating both quantitative and qualitative assessments of staining patterns.

Therefore, this study aimed to determine whether the immunolocalization and immunoexpression of MMP-2 and TIMP-2 differ significantly in grafted kidneys depending on the presence or absence of fibrosis. To address this objective, we analyzed the immunoexpression of MMP-2 in renal biopsy specimens.

Our results demonstrated that the immunohistochemically positive area for MMP-2 was significantly smaller in fibrotic kidneys compared with control biopsies. Conversely, the immunohistochemically negative area for MMP-2 was significantly larger in the control group than in fibrotic kidneys. Overall, these findings suggest that fibrotic kidneys exhibit a reduced sensitivity to immunohistochemical staining for MMP-2. Our findings, however, are in contrast to Yan et al. [23]. On the other hand, it has been shown that MMP-2 immunoexpression tended to decrease, along with the increase in the pathological grade of interstitial fibrosis or tubular atrophy. For instance, Mengel et al. have observed in patients suffering from renal fibrosis a significantly lower expression of mRNA for MMP-2 compared to controls [24]. According to the available data, in the early stage of kidney fibrosis, MMP-2 promotes ECM production, thus accelerating the development of kidney fibrosis. In the advanced stage, MMP-2 activity decreases, leading to reduced ECM degradation and making it difficult to alleviate kidney fibrosis [24]. The mechanisms underlying the decrease in MMP-2 activity in advanced stages of fibrosis remain unclear and require further investigation. One possible explanation is the presence of hypoxia and altered endocytic processes, which may influence the expression and activity of the regulatory molecules involved in MMP-2 activation. These changes could disrupt the balance between matrix synthesis and degradation, thereby contributing to fibrosis progression.

In the present study, we observed an increased MMP-2/TIMP-2 ratio in the fibrotic group compared with the control group. The detailed analysis indicated that this alteration was primarily attributable to differences in the relative distribution of TIMP-2-positive areas within the renal tissue compared with MMP-2-positive areas. Notably, the overall immunoexpression of both markers was reduced in fibrotic kidneys, with an approximate 50% decrease in TIMP-2 and a 25% decrease in MMP-2 compared with controls.

The comparatively smaller reduction in MMP-2 expression suggests a relatively diminished inhibition, which may facilitate enhanced extracellular matrix remodeling during fibrosis progression. This imbalance between MMP-2 and TIMP-2 may therefore contribute to the dysregulated ECM turnover characteristics of fibrotic kidney tissue. Our result is consistent with the available literature, suggesting an imbalance in MMP/TIMP in chronic kidney disease, where the dominance of the activity of MMP-2 over TIMP is associated with the progression of fibrosis [25].

Furthermore, we have analyzed the immunopositive areas of renal biopsies in terms of the intensity of the positive reaction. We have observed that the area of strong positive immunoexpression (++) for MMP-2 was significantly higher in the control group compared to grafted kidneys with fibrosis. Additionally, while assessing the potential correlation between tested variables, it was found that, in the control group, a weak positive reaction (+) of MMP-2 was indeed positively correlated with a strong positive immunoexpression, as well as with a negative immunoexpression (−). This partly goes against the results of Wong et al. [26], who observed that all kidney biopsies had the same level of staining in the tubular epithelium, without any significant difference between the control group and patients with chronic humoral rejection. In contrast, distinctive staining patterns were observed in the glomeruli, indicating a different distribution of MMP-2 [26]. Another interesting study conducted on patients suffering from progressive tubulointerstitial fibrosis, with stages three and four, showed that the median urinary MMP-2/creatinine ratio was significantly higher than that in the controls [27].

For a deeper analysis, we have checked whether our current results in terms of immunoexpression for MMP-2 have any correlation with each other. Indeed, we have observed in the fibrotic group a correlation between weak positive (+) and strong positive (++) as well as very strong positive (+++), and negative immunoexpression (−). Moreover, strong positive immunoexpression (++) has been found to correlate with very strong positive (+++) and with negative (−) immunoexpression. What is more, very strong positive immunoexpression (+++) was correlated with negative immunoexpression (−). These findings, however interesting, are hard to discuss due to the lack of appropriate literature. Nevertheless, it might be a consequence of very complex changes that occur in the ECM in fibrotic tissue.

Further current analysis was also conducted regarding the immunostaining intensity of the renal biopsies for TIMP-2, since MMPs and TIMPs play a central role in maintaining the homeostasis, remodeling, and accumulation of the ECM [28]. Commonly, the scientific literature focuses on serum creatinine while assessing the condition of the kidneys, both native and grafted. This parameter is indeed crucial in the diagnosis of kidney failure; however, it is unfortunately not an optimal biomarker for the timely evaluation of renal damage due to its certain latency in increasing after the occurrence of kidney damage. Therefore, preventive measures may not have a sufficient effect, since the damage has already occurred, and it is irreversible [29].

We have currently noticed that the strong positive immunoexpression (++) for TIMP-2 in the control group was significantly higher when compared with the fibrotic group. This stands in contrast to Schanz et al. [29]. One of the possible explanations for this discrepancy may be the fact that TIMP-2 biology is complex. On the one hand, TIMPs are inhibitors of MMPs; on the other hand, they contribute to the activation of MMP-2 via the MT1-MMP. Hence, fluctuations in TIMPs do not always correlate with an increase in fibrosis [10]. For a broader analysis of the topic, we assessed the correlations between the immunoexpressions of TIMP-2. In the control group, we have indeed revealed that a weak positive immunoexpression (+) correlates with a strong positive immunoexpression (++), and strong positive immunoexpression (++) correlates with very strong positive immunoexpression (+++). Interestingly, within the fibrotic group, no correlations between the tested variables were found. Unfortunately, as already mentioned, the findings on TIMP-2 expression in human kidney biopsies of renal disease are scarce in terms of data. It seems that the results vary depending on the model of the research, the stage of a certain kidney disease, and the technique that has been applied to assess the results.

This study is not free from limitations. First, it is a single-center investigation with relatively small sample sizes, which may limit the generalizability of the findings. Although larger cohorts would increase statistical power and reliability, it should be emphasized that our material consists of human graft tissue, which is rare and inherently limited in availability.

Another limitation is the absence of complementary analyses of gene and protein expression for key fibrosis-related markers. A more comprehensive evaluation would include an assessment of profibrotic mediators such as TGF-β1, IL-4, IL-13, TNF-α, MCP-1, IL-6, and IL-8, as well as altered collagen deposition within the tissue and relevant circulating biomarkers. The inclusion of these parameters would provide a broader mechanistic understanding of extracellular matrix remodeling in graft fibrosis.

Nevertheless, we consider this study preliminary. Further investigations involving larger, multicenter cohorts and more advanced molecular analyses, including gene expression profiling and quantitative protein assays, are warranted to expand upon our findings.

## 5. Conclusions

The remodeling of the extracellular matrix is more prominent in the fibrotic renal graft in comparison to the control tissue, since the MMP-2/TIMP-2 ratio is higher in the fibrotic renal grafts when compared to the control group. It seems that the MMP-2/TIMP-2 ratio, together with functional parameters, is a good indicator that reflects clinical alterations. Moreover, this parameter correlates with our morphometrical studies.

Modern and standardized methods based on digital analysis undoubtedly appear to be a milestone in modern histopathology.

## Figures and Tables

**Table 1:** Characteristics of patients presented as median value (Me), minimum (Min), and maximum value (Max).

	*n*	Me	Min	Max
Time from Tx (months)	44	69	12	182
Age (years)	44	42	24	71
eGFR on last visit (mL/min/1.73m^2^)	44	36	15	89
Protein in urine (mg/dL)	44	0	0	865.7
Time of dialysis before Tx (months)	44	15	6	102
CIT (min)	44	1260	72	2100
PRA (%)	44	3	0	56
ZENITH eGFR (mL/min/1.73m^2^)	44	31.0	98.0	118.0
Cause of Renal Failure				
Chronic glomerulonephritis	14			
Diabetes	7			
Unknown	7			
Autosomal dominant polycystic kidney disease	6			
Inflammatory diseases	4			
Hypertension	3			
Other	4			
SEX	Female: 21		Male: 23	

Tx—renal transplantation; ZENITH eGFR—highest estimated glomerular filtration rate in the first 6 months after Tx.

**Table 2:** The impact of renal fibrosis on the diameter of the renal corpuscle (A), the diameter of the glomerulus (B), the width of the urinary space (*), and the height of the epithelial lining of the proximal (C) and distal (D) convoluted tubules. Data presented as medians and IQRs, including *p*-value for the Kruskal–Wallis test and correlation evaluated using the Spearman test. All measurement results are presented in mm.

Variable	Control (*n* = 22)	Fibrosis (*n* = 22)
Renal corpuscle (A)	122.1 * # (100.8; 142)	165.45 & @ (140.3; 191.9)
Glomerulus (B)	110.7 * (87.8; 126)	115.95 @ $ (86.6; 134.7)
Urinary space (*)	11.7 # (7.7; 16.5)	47.95 & $ (30.4; 69.9)
Proximal convoluted tubule (C)	13.2 (11.7; 15.1)	12.5 (11.1; 14.6)
Distal convoluted tubule (D)	8.4 (7.4; 9.4)	6.4 (5.6; 7.4)

Correlation between tested variable with value of * = 0.952; # = 0.192; & = 0.354; @ = 0.69; $ = −0.28.

**Table 3:** The positive immunoexpression areas for MMP-2 and TIMP-2, with the MMP-2/TIMP-2 ratio in the control and the fibrotic group.

Variable	Control (*n* = 22)	Fibrosis (*n* = 22)
MMP-2 (mm^2^)	1753.59	1321.08
TIMP-2 (mm^2^)	3382.8	1685.2
MMP-2/TIMP-2 ratio	0.51	0.784

**Table 4:** The immunoexpression for MMP-2 is expressed in mm^2^ and the percentage of the analyzed area in the control and the fibrotic group. Data presented as medians and IQRs (where Q3 is the upper quartile, and Q1 is the lower quartile), including *p*-value for the Kruskal–Wallis test and correlation evaluated using the Spearman test.

Variable	Control (*n* = 22)			Fibrosis (*n* = 22)			*p*-Value (Kruskal–Wallis)
	Median	Q1	Q3	Median	Q1	Q3	
+ (mm^2^/%)	61.35/44.82 * #	1.38/25.49	253/61.74	42.68/33.27 & @ $	0.043/26.06	122.55/41.48	0.27
++ (mm^2^/%)	20.65/12.09 *	0.28/3.04	40.6/20.08	1.71/8.35 & ^ ~	0.04/0.002	22.68/13.1	0.02
+++ (mm^2^/%)	0.18/0.25	0.001/0.01	0.35/0.67	0.008/0.41 @ ^ !	0.002/0.01	0.45/3.47	0.47
− (mm^2^/%)	364.85/42.17 #	285.7/25.42	1040/53.99	306.1/55.24 $ ~ !	51.75/42.61	648.2/67.8	0.2

(−): negative expression; (+): weak expression; (++): strong expression; (+++): very strong expression. Correlation between tested variable with value of * = 0.6; # = 0.71; & = 0.9; @ = 0.68; $ = 0.66; ^ = 0.88; ~ = 0.78; ! = 0.57.

**Table 5:** The immunoexpression for TIMP-2 expressed in mm^2^ and the percentage of analyzed area in the control and the fibrotic group. Data presented as medians and IQRs (where Q3 is the upper quartile, and Q1 is the lower quartile), including *p*-value for the Kruskal–Wallis test and correlation evaluated using the Spearman test.

Variable	Control (*n* = 22)			Fibrosis (*n* = 22)			*p*-Value (Kruskal–Wallis)
	Median	Q1	Q3	Median	Q1	Q3	
+ (mm^2^/%)	346.4/28.5 *	54.5/19.67	488.4/32.17	173.2/28.54	88.2/24.68	318.3/30.68	0.41
++ (mm^2^/%)	222.1/23.32 * #	111.6/20.97	424.5/25.9	96.95/20.95	75.9/16.46	145.7/22.75	0.01
+++ (mm^2^/%)	128.4/11.08 #	18.8/8.94	150.5/13.62	38.41/9.65	25.4/7.71	111.55/16.01	0.19
− (mm^2^/%)	482/35.94	82/31.07	908/38.34	124.45/39.19	98.2/32.78	338.85/41.4	0.25

(−): negative expression; (+): weak expression; (++): strong expression; (+++): very strong expression. Correlation between the tested variable with values of * = 0.67; # = 0.53.

## Data Availability

The original contributions presented in this study are included in the article. Further inquiries can be directed to the corresponding author.

## Abbreviations

The following abbreviations are used in this manuscript:

CKD	Chronic kidney disease
ECM	Extracellular matrix
MMPs	Metalloproteinases
TIMPs	Tissue inhibitors for MMPs
H&E	Hematoxylin and eosin
IQR	Interquartile range
